# Supplementary material for: Analysis of influencing factors on review efficiency of multidisciplinary scientific research projects using DEMATEL with a 5-point scale
Source: PLoS One. 2024 Dec 12;19(12):e0315349. doi: 10.1371/journal.pone.0315349 (PMC11637369; doi:10.1371/journal.pone.0315349)
Supplement: S1 Questionnaire — (PDF) [file pone.0315349.s001.pdf]

<u>Questionnaire on reviewing multidisciplinary scientific research project</u>

Instructions: Please rate each factor based on its influence on the review efficiency of multidisciplinary scientific research projects. Use a scale from 1 (Unimportant) to 5 (Very Important). Additionally, each question includes a brief description to clarify its scope.

1. Unimportant
2. Relatively Low
3. General
4. Relatively High
5. Very Important

Part 1: Project Factors

1. Scope of Project Knowledge:
Description: Assess how the breadth and depth of knowledge within the project affect its review process.
Rating: ____

2. Interdisciplinary Collaboration:
Description: Evaluate the impact of collaboration between different disciplines on the review efficiency.
Rating: ____

3. Resource Allocation:
Description: Consider how the distribution and availability of resources influence the review process.
Rating: ____

4. Market Factors:
Description: Rate the effect of market-related factors, such as demand and relevance, on the review efficiency.
Rating: ____

5. Time factors:
Description: Refer to the adherence to project timelines and milestones, indicating the efficiency and effectiveness of project management and its impact on the review process.
Rating: ____

6. Social factors:
Description: Encompass the societal impacts, stakeholder interests, and ethical considerations of a project, reflecting its holistic approach and broader implications beyond technical and financial aspects.
Rating: ____

7. Psychological factors:
Description: Involve the morale, motivation, and mental well-being of the project team, significantly influencing project outcomes and the team's ability to proactively address challenges.
Rating: ____

Part 2: Review Factors

1. Review Criteria:
Description: Reflect on how clearly defined and relevant criteria affect the objectivity and consistency of reviews.

Rating: ____

2. Stakeholder Involvement:
Description: Determine the importance of involving stakeholders in the review process for comprehensive insights.
Rating: ____

3. Psychological Factors:
Description: Assess the impact of psychological aspects, such as motivation and perception, on the review process.
Rating: ____

4. Review Methodology:
Description: Evaluate the influence of the methodologies and techniques used in the review process.
Rating: ____

5. Feedback Mechanisms:
Description: Consider the role of feedback in shaping the review process and its outcomes.
Rating: ____

6. Transparency and Openness:
Description: Rate the impact of transparency in criteria, methodologies, and feedback on the review process.
Rating: ____

7. Continuous Monitoring:
Description: Assess the importance of ongoing monitoring and periodic evaluations in the review process.
Rating: ____

Additional Questions
1. In your opinion, which factor(s) most significantly impact the efficiency of reviews in multidisciplinary projects?
Description: Share your opinion on which factors most significantly impact the efficiency of reviews in multidisciplinary projects.

Response: _______________

2. Do you have any suggestions for improving the review process in multidisciplinary scientific research projects?
Description: Provide any suggestions you have for improving the review process in multidisciplinary scientific research projects.

Response: _______________
