# Supplementary material for: Analysis of influencing factors on review efficiency of multidisciplinary scientific research projects using DEMATEL with a 5-point scale
Source: PLoS One. 2024 Dec 12;19(12):e0315349. doi: 10.1371/journal.pone.0315349 (PMC11637369; doi:10.1371/journal.pone.0315349)
Supplement: Attachment — Submitted filename: Response to Reviewers.docx [file pone.0315349.s002.docx]

**Responses to Editor and Reviewers**

Manuscript ID: PONE-D-23-29808

Title: Analysis of influencing factors of review efficiency of multidisciplinary scientific research projects based on improved DEMATEL model

Dear Editor and Reviewers,

We would like to express our sincere appreciation for the time and effort the reviewers have taken in evaluating our manuscript, "*Analysis of influencing factors of review efficiency of multidisciplinary scientific research projects based on improved DEMATEL model*". We find the reviewers' comments insightful and helpful in enhancing the quality of the manuscript.

To address the reviewers’ comments, we have carefully revised our manuscript and believe that these modifications have substantially improved the manuscript. We have provided a point-by-point response to the comments below. In addition, we have uploaded a marked copy of the manuscript as a supplemental file to clearly highlight the changes made in response to the reviewers' comments.

Thank you once again for considering our work for publication in *PLoS One*. We believe that our manuscript has been significantly improved and hope that it is now suitable for publication.

I look forward to hearing from you regarding our submission.

Sincerely yours,

Yana Xiao

Senior engineer/Director

Guangdong Science & Technology Infrastructure Center

Email: ynaxiao@163.com

**Response to Editor**

**Comment 1:** I believe there is a word missing at the beginning of line 35.

**Response:** Thanks for your reminder. This error was caused by a misoperation when we generated citations, and we are very sorry for this. In the revised manuscript, we have added the subject and references at the beginning of line 35 as follows:

“*Purvis et al. [11] reviews multidisciplinary research projects present a unique set of challenges that stem from the inherent complexity and breadth of such endeavors.*”

**Comment 2:** in my opinion part "2.1 Previous studies for reviews multidisciplinary projects" is too focused on AI. It needs either more adequate title or slight change of focus so that AI will not dominate the discussion here.

**Response:** Thanks for your suggestions. Indeed, the original review of the current research status was not comprehensive enough and overemphasized the AI part. In the revised manuscript, we have added a more comprehensive review of previous studies for reviews of multidisciplinary projects. The supplementary content is as follows:

“*Historically, the value of multidisciplinary research has been well-recognized. Bentrem's sabbatical insights at the Naval Research Laboratory [21] underscored the unique perspectives and solutions that such approaches bring to scientific research. More recently, Beck et al. [22] delved into the challenges of interdisciplinary collaboration, particularly between computer science and social science. Their proposed tool for multidisciplinary dialogue emphasizes the co-construction of models, with applications evident in areas such as post-earthquake human behavior modeling. A study by Pan et al. [23] in small biotechnology firms revealed that project success in multidisciplinary settings is influenced by the project knowledge scope and the project manager's experience. The study underscores the importance of skilled project management in navigating the complexities of multidisciplinary projects. Ma et al. [24] presented a model for dividing responsibilities in multidisciplinary teams. This model, applied in an OEM company, demonstrated improved efficiency and reduced conflict, highlighting the importance of clear role delineation in multidisciplinary settings. The role of leadership in project management education, as discussed by Mazzetto [25], emphasizes the need for project managers to possess strong leadership skills to guide diverse teams effectively. Burnette et al. [26] identified five major themes crucial for successful data management in multidisciplinary projects, including intentional staffing and iterative improvement, which are essential for managing complex data landscapes. Studies by Mazzetto [27] and Urton et al. [28] focus on the integration of practical experience in project management education, suggesting that real-world experience is critical for preparing future project managers. Previous studies in multidisciplinary project management emphasize the importance of project knowledge scope, experienced leadership, clear role delineation, effective data management, and practical experience in education for successful project outcomes in diverse and complex environments.*”

**Comment 3:** reference in line 111 is missing.

**Response:** We apologize for missing key citations that affect the readability of the paper. This problem was caused by incorrectly imported references, and we have added the corresponding references in the revised manuscript. The revised content is as follows:

“*The DEMATEL method has emerged as a pivotal tool in the domain of complex systems and decision-making [32,33]*”

**Comment 4:** speaking of adaptability of the DEMATEL method, you should include some references or examples (lines 143-145).

**Response:** We apologize for the lack of citation of corresponding references in the previous statement. In the revised manuscript, we have added the following citations to existing work to support our statements:

“*The DEMATEL method stands as a testament to the evolution of decision-making tools, adeptly handling the complexities of modern systems [41,42].*”

**Comment 5:** how do you define "reviews efficiency"? It is crucial for understanding the study.

**Response:** Thanks for your question. In our study, we define "review efficiency" as the effectiveness and expediency of the review process in evaluating multidisciplinary scientific research projects. This encompasses the ability to provide comprehensive, actionable feedback within a reasonable timeframe, thereby facilitating informed decision-making and iterative improvements in the project. Our definition aligns with the structured, systematic methodologies tailored to multidisciplinary projects, as highlighted in our manuscript (Page 5). The efficiency of the review process is crucial as it directly impacts the project's progression and quality, ensuring that the multidisciplinary nature of the projects does not hinder their successful completion and implementation.

We apologize for the confusion caused by not providing a clear definition of “reviews efficiency” in the original manuscript. In the revised manuscript, we have revised the last paragraph of the *Introduction* section as:

“*The objective of this study centers on addressing the complexities and challenges inherent in reviews multidisciplinary scientific research projects. Recognizing the pivotal role of collaborative research across academic sectors in driving innovation and addressing multifaceted challenges, the study seeks to elucidate the factors influencing the review process of such interdisciplinary endeavors. Given the significance of efficient review mechanisms to the sustainable progression of the scientific research domain, the study endeavors to enhance review efficiency, ensuring the seamless development and improved quality of research projects. Among them, review efficiency is defined as the effectiveness and convenience of the review process in evaluating multidisciplinary scientific research projects [18]. This includes the ability to provide comprehensive, actionable feedback within a reasonable time frame, thereby promoting informed decision-making and iterative improvements within projects. This definition is consistent with a structured, systematic approach tailored to multidisciplinary projects and is critical to the efficiency of the review process as it directly affects the progress and quality of the project, ensuring that the multidisciplinary nature of the project does not hinder Its successful completion and implementation. To achieve this, the study introduces a systematic indexing framework to identify factors influencing the appraisal of multidisciplinary efforts. This framework is further enriched by expert-driven questionnaires, capturing domain-specific insights to determine the significance and interplay of these factors. Employing an improved Decision Making Trial and Evaluation Laboratory (DEMATEL) model, the study distills this data to pinpoint key influencing factors that can bolster review efficiency for multidisciplinary projects. The culmination of these efforts aims to provide pragmatic strategies and policy guidance, arming institutional entities and program leaders with the requisite tools to optimize the review process for multidisciplinary scientific research projects.*”

[18] Dekkers, R., Carey, L., & Langhorne, P. (2022). *Making literature reviews work: A multidisciplinary guide to systematic approaches*. Springer.

**Comment 6:** were "project factors" and "reviews factors" suggested by experts or Authors?

**Response:** Thanks for your question. The "project factors" and "reviews factors" were derived through a comprehensive methodology. Initially, we conducted a thorough literature review using specific keywords to identify preliminary factors. This was followed by expert consultations in the field of scientific research project management. These experts played a crucial role in refining and adjusting the preliminary elements, leading to the finalization of the indicator framework that categorizes these factors into "project factors" and "reviews factors" (Page 5 of the manuscript). This approach ensured that the factors identified were not only grounded in existing literature but also validated and enriched by professional expertise in the field.

**Comment 7:** why mean was used in the study? Have you considered using median or mode?

**Response:** Thanks for your question. The decision to use the mean as the measure of central tendency was driven by its appropriateness for the data characteristics and the analysis method employed. The mean was chosen because it provides a balanced central point that reflects the average influence of factors in the DEMATEL method, which is particularly effective in handling continuous data and symmetric distributions often encountered in such studies. While median and mode are valuable measures, they were not considered optimal for our analysis due to the nature of our data and the specific requirements of the DEMATEL methodology. The mean offers a more nuanced understanding of the central tendency in the context of our study, where the precise average impact of factors is crucial for the subsequent analysis stages.

**Comment 8:** "So far, this method has been used in computer science, economics and management, social sciences and other fields to solve practical scientific problems." - lines 218-219 - references or examples are needed here.

**Response:** Thanks for your suggestions. We apologize for not providing sufficient supporting references. In the revised manuscript, the references have been supplemented as:

“*So far, this method has been used in computer science [49], economics and management [50], social sciences [51], and other fields to solve practical scientific problems [52,53].*”

[49] Chaker, F., El Manouar, A., & Idrissi, M. A. J. (2015). Towards a system dynamics modeling method based on DEMATEL. *International Journal of Computer Science & Information Technology*, 7(2), 27.

[50] Sharma, M., Joshi, S., & Kumar, A. (2020). Assessing enablers of e-waste management in circular economy using DEMATEL method: An Indian perspective. *Environmental Science and Pollution Research*, 27(12), 13325-13338.

[51] Braga, I. F., Ferreira, F. A., Ferreira, J. J., Correia, R. J., Pereira, L. F., & Falcão, P. F. (2021). A DEMATEL analysis of smart city determinants. *Technology in Society*, 66, 101687.

[52] Yazdi, M., Khan, F., Abbassi, R., & Rusli, R. (2020). Improved DEMATEL methodology for effective safety management decision-making. *Safety science*, 127, 104705.

[53] Koca, G., Egilmez, O., & Akcakaya, O. (2021). Evaluation of the smart city: Applying the dematel technique. *Telematics and Informatics*, 62, 101625.

**Comment 9:** you refer to "improved DEMATEL", but do not explain how it is improved as compared with the original version.

**Response:** Thanks for your question. The improvement of the DEMATEL method was achieved through several key enhancements. Firstly, we incorporated a five-point scoring system to assess the degree of influence of each factor, ranging from 'unimportant' to 'very important.' This modification allowed for a more nuanced and precise evaluation of the factors' impacts. Additionally, we introduced a step of scoring the influence intensity of each factor itself, which improved the accuracy of the reviews. These enhancements were designed to address the limitations of the traditional DEMATEL method, particularly in terms of capturing the subtleties and complexities inherent in multidisciplinary project reviews. The improved DEMATEL model thus provided a more refined and accurate analysis of the influencing factors and their interrelationships, enhancing the overall robustness and applicability of the method in our study.

Indeed, the lack of description of the differences between improved DEMATEL and traditional DEMATEL methods can cause confusion for readers. In the revised manuscript, we added a clarification on the improvements of improved DEMATEL at the end of Section 3.2, explaining the main contribution of this study and the differences compared with existing research as follows:

“*The traditional DEMATEL method, known for its ability to visualize and quantify complex causal relationships within systems, has been adapted to better suit the intricacies of multidisciplinary project reviews. The improved DEMATEL method in this study introduces a novel five-point scoring system, allowing for a more granular assessment of the influence of each factor, ranging from 'unimportant' to 'very important'. This enhancement addresses the need for a more nuanced understanding of the varying degrees of influence among factors. Additionally, the study incorporates a new step of scoring the influence intensity of each factor individually, further refining the accuracy of the review process. These improvements in the DEMATEL method provide a more precise and detailed analysis of the factors influencing the efficiency of reviews in multidisciplinary scientific research projects, thereby offering deeper insights and more actionable outcomes.*”

**Comment 10:** in my opinion questionnaire should be attached as a supporting file.

**Response:** Thanks for your suggestions. We agree that providing the questionnaire as an accessible document would be beneficial for readers and reviewers who wish to understand the methodology in greater detail. Accordingly, we have attached the questionnaire as a supplementary file to our manuscript submission. This addition will offer comprehensive insights into the structure and content of the questionnaire, thereby enhancing the transparency and reproducibility of our research.

**Comment 11:** I do not understand the notation in line 329.

**Response:** Thanks for your reminder. We apologize for a clerical error that resulted in the incorrect use of a symbol. The original manuscript was written using LaTeX, and we mistakenly used ">" directly in the draft, which caused LaTeX not to recognize it. In the revised manuscript, we have used "\textgreater" instead. The modified corresponding symbols can be displayed normally as follows:

“*In the resultant group, the centrality sequence is F11*$>$*F12*$>$*F10*$>$*F8*$>$*F14*$>$*F13*$>$*F5*$>$*F9.*”

**Comment 12:** I would refrain from using word "significant" since it suggest statistical test were run.

**Response:** Thanks for your suggestions. To avoid any potential misunderstanding, we have revised the manuscript to replace "significant" with terms like "notable" or "substantial," which more accurately reflect the qualitative nature of our analysis. This change will ensure that the language in our manuscript aligns with the intended meaning and avoids any implication of statistical testing where it was not employed.

Thanks again for your comments, which have truly improved the readability and quality of this manuscript. We hope our responses address your concerns.

**Response to Reviewer #1**

**Comment 1:** The theoretical basis for the mechanisms presented in this study is very weak and unconvincing. The novelty of this manuscript is very inadequate. There are many similar studies. In addition, the research purpose and research framework of the manuscript are unclear. There are unaddressed issues in the paper that bear on the central conclusions of the paper.

**Response:** Thanks for your comments. We acknowledge your concerns and provide the following clarifications:

**Theoretical basis:** Our study is grounded in a robust theoretical framework that integrates interdisciplinary research methodologies. We have utilized the DEMATEL method, a proven approach in complex systems analysis, and enhanced it for the specific context of multidisciplinary project reviews. This methodological choice is supported by a comprehensive literature review, ensuring a solid theoretical underpinning.

**Novelty:** The novelty of our study lies in the application of the improved DEMATEL model to the specific field of multidisciplinary project reviews, a relatively unexplored area in project management research. Our approach offers new insights into the dynamics of such reviews, addressing a gap in existing literature.

**Research purpose and framework:** The primary objective of our research is to identify and analyze the factors influencing the efficiency of reviews in multidisciplinary scientific research projects. We have clearly outlined this purpose in the introduction and developed a systematic indexing framework to categorize these factors. This framework, coupled with expert-driven questionnaires, provides a comprehensive analysis of the influencing factors, contributing to the field's understanding of multidisciplinary project reviews.

**Addressing unaddressed issues:** We recognize the importance of addressing all pertinent issues in our research. To this end, we have thoroughly examined the interdependencies and impacts of various factors on review efficiency, as detailed in our methodology and results sections.

We hope this response adequately addresses your concerns and clarifies the aspects of our manuscript.

**Response to Reviewer #2**

**Comment 1:** The manuscript doesn’t give any information on the collection of documents that was used for the search of indicators (Elsevier’s ScienceDirect, SpringerLink, open access publications of other science publishers?), in which years these documents were published, how many documents were browsed. The reader doesn’t know either who are the experts that were consulted on the influencing factors or how many experts were involved.

**Response:** Thanks for your suggestions. We acknowledge the need for clarity in these areas and provide the following details:

For the literature review, we employed a systematic approach to source documents. We used keywords such as “multidisciplinary & influencing factors,” “scientific research projects & influencing factors,” and “project reviews & indicators” to search across various academic databases, including Elsevier’s ScienceDirect, SpringerLink, and open access publications. The documents spanned publications from the last two decades, ensuring a comprehensive and current perspective. The initial search yielded a substantial number of documents, which were then meticulously screened to remove duplicates and retain the most relevant and impactful studies.

The selection of experts was a critical component of our study. We engaged a panel of experts from diverse backgrounds, including educational institutions, industries, and research entities, primarily based in Pakistan. These experts were not only well-qualified but also had significant experience in project management and had served on various selection committees. The number of experts involved provided a broad range of insights, enhancing the validity of our findings.

Accordingly, we modified Section 3.1 Development of the indexing framework of the manuscript to:

“***3.1 Development of the indexing framework***

*To develop a comprehensive indexing framework for the review efficiency of multidisciplinary scientific research projects, a meticulous document collection and expert consultation process has been employed. The framework was designed to capture the multifaceted nature of such projects and provide a structured approach to evaluating their review efficiency.*

*For the document collection process, a literature search was conducted across several academic databases, including Elsevier’s ScienceDirect, SpringerLink, and various open access publications. this study focus on documents published in the last two decades to ensure the relevance and currency of our data. The search strategy involved using specific keywords such as “multidisciplinary \& influencing factors,” “scientific research projects \& influencing factors,” and “project reviews \& indicators.” This approach yielded a substantial number of documents, which were then rigorously screened to remove duplicates and retain only the most pertinent studies. This process also ensured a diverse and comprehensive collection of literature, forming the basis of our indexing framework.*

*For the expert consultation, the refinement of the indexing framework was further enhanced through consultations with a panel of experts. These experts were selected based on their qualifications, experience, and contributions to the field of project management and multidisciplinary research. They represented a mix of academic and industry perspectives, providing a well-rounded view of the factors influencing review efficiency. The number of experts involved in the consultation process was carefully chosen to ensure a broad range of insights while maintaining the manageability of the consultation process.*

*For the framework development, the indexing framework was developed through an iterative process, combining insights from the literature review and expert consultations. This process involved categorizing the influencing factors into distinct groups, such as project factors and review factors, and then evaluating their interrelationships and impact on review efficiency. The framework was designed to be adaptable, allowing for the incorporation of new factors and insights as the field of multidisciplinary project management evolves.*

*This comprehensive approach to developing the indexing framework ensures that it is grounded in both theoretical knowledge and practical insights, making it a valuable tool for evaluating the review efficiency of multidisciplinary scientific research projects.*”

We hope this response adequately addresses your concerns and adds clarity to the methodology of our research.

**Comment 2:** The inclusion of factors such as F3, F4, F6 and F12 suggests that the authors are interested in the applied research rather than in the theoretical one, although earlier they mention such outcomes of the research as “the emergence of innovative theoretical constructs” (lines 20-21) and pay attention to the possibility that “potential outcomes and impacts of the research are not immediately tangible” (lines 49-50). The legibility of the paper would increase if the authors clarified whether they are interested in applied research only (without referring to the theoretical research or mentioning this kind of research with a due disclaimer) or in both applied and theoretical research (in such case the applicability of factors F3, F4, F6 and F12 should be explained).

**Response:** Thanks for your insightful comment. We appreciate the opportunity to clarify our research focus and the applicability of these factors.

**Applied vs. Theoretical Research:** Our study aims to bridge the gap between theoretical constructs and their practical applications in multidisciplinary project reviews. While we acknowledge the emergence of innovative theoretical constructs (lines 20-21) and the potential for non-tangible outcomes (lines 49-50), our primary focus is on applied research. This focus is driven by the need to provide actionable insights and strategies that can be directly implemented in the review processes of multidisciplinary projects.

**Applicability of Factors:** The inclusion of factors such as F3, F4, F6, and F12 is rooted in their relevance to both theoretical understanding and practical applications in multidisciplinary project reviews. For instance:

- F3 (Profitability): This factor is included to highlight the importance of clear objectives and structured approaches in both theoretical and applied research, facilitating streamlined review processes.
- F4 (Market Factors): Understanding market demands and trends is crucial for aligning research projects with practical needs, ensuring their relevance and potential for success.
- F6 (Social Factors): Projects that consider societal impacts and ethical considerations demonstrate a holistic approach, bridging theoretical concepts with real-world implications.
- F12 (Stakeholder Involvement): Involving stakeholders provides valuable perspectives that enrich both the theoretical framework and practical applications of the research.

In summary, our study encompasses both theoretical and applied research aspects, with a stronger emphasis on the latter. The inclusion of the aforementioned factors is intended to enhance the practical utility of our findings while maintaining a connection to theoretical underpinnings.

**Comment 3:** The factors F5 and F7, and to the lesser extent F10 and F14, indicate that the focus of the study is the interim review of the research projects rather than the projects that apply for funding. It would be useful to clarify this issue.

**Response:** Thanks for your

**Comment 4:** The experts provided also the scoring tables that were used to calculate the direct influence matrix and the standardized matrix. Then the total influence matrix was computed and the indicators of centrality/prominence and causality were evaluated. According to these indicators the reviewer expertise appeared to be the least significant factor, although earlier it was assumed that the requisite expertise of reviewers is needed (line 37), and in the description of factor F9 the expertise and experience of reviewers was assessed as being paramount (lines 190-191). Such a low rank of this factor requires some additional comment and explanation.

**Response:** Thank you for your observation regarding the inclusion of factors F5 (Time Factors), F7 (Psychological Factors), F10 (Feedback Mechanisms), and F14 (Continuous Monitoring) in our study. We appreciate the opportunity to clarify the focus of our study. Our study primarily focuses on the interim review of multidisciplinary scientific research projects rather than the initial funding application process. This focus is reflected in the inclusion of factors that are more relevant to the ongoing evaluation and improvement of projects during their execution phase. Relevance of Selected Factors:

- F5 (Time Factors): The inclusion of time factors emphasizes the importance of adhering to timelines and milestones in project management, which is a critical aspect of interim reviews.
- F7 (Psychological Factors): Psychological factors are included to highlight the impact of team morale and motivation on project outcomes, which are often evaluated during interim reviews.
- F10 (Feedback Mechanisms): Effective feedback mechanisms are essential for iterative improvements in projects, a key component of interim reviews.
- F14 (Continuous Monitoring): Continuous monitoring and periodic evaluations align with the concept of interim reviews, allowing for ongoing adjustments and improvements in projects.

The inclusion of these factors underscores our study's emphasis on the dynamic and ongoing nature of project reviews. Interim reviews are crucial for assessing progress, identifying areas for improvement, and making necessary adjustments, which are essential for the successful completion of multidisciplinary projects.

In summary, our study is geared towards understanding and enhancing the efficiency of interim reviews in multidisciplinary scientific research projects. The factors F5, F7, F10, and F14 are specifically chosen to reflect this focus, providing insights into the various elements that influence the effectiveness of these reviews. We hope our clarification addresses your concerns.

**Comment 5:** The definition of factor F8, the choice of properly structured, systemic review methodology that is tailored to multidisciplinary projects (lines 186-188), seems vague. The declared objectives of the study raise the expectations that specification of the review methodology will be the outcome of the analysis rather than one of generally defined factors.

**Response:** Thanks for your reminder. We apologize for the confusion the lack of clarification has caused you. For F8 we have the following clarifications:

- Role of Factor F8: Factor F8, 'Review Methodology,' is included in our study to emphasize the importance of selecting and implementing a review methodology that is specifically adapted to the unique challenges and characteristics of multidisciplinary projects. This factor is crucial because multidisciplinary projects often span various domains and require a review approach that can effectively address their diverse aspects.
- Definition and Specificity: We acknowledge that the initial definition of F8 in our manuscript may appear general. However, the intention was to highlight the need for a flexible and adaptable review methodology that can be tailored to the specific requirements of different multidisciplinary projects. The factor is not meant to prescribe a specific methodology but rather to underscore the importance of choosing a methodology that is congruent with the project's multidisciplinary nature.
- Outcome of the Analysis: While the study aims to identify factors influencing review efficiency, it does not necessarily provide a detailed specification of the review methodologies themselves. Instead, F8 serves as a reminder of the critical role that the choice and adaptation of review methodologies play in the overall efficiency of project reviews.

In summary, factor F8 is included in our study to highlight the significance of selecting a review methodology that aligns with the multidisciplinary nature of the projects under review. It is a call for adaptability and specificity in review processes, rather than a detailed prescription of particular methodologies.

Thanks again for your comments, which have truly improved the readability and quality of this manuscript. We hope our responses address your concerns.
