# Supplementary material for: Analysis of influencing factors on review efficiency of multidisciplinary scientific research projects using DEMATEL with a 5-point scale
Source: PLoS One. 2024 Dec 12;19(12):e0315349. doi: 10.1371/journal.pone.0315349 (PMC11637369; doi:10.1371/journal.pone.0315349)
Supplement: Attachment — Submitted filename: Response to Reviewers.docx [file pone.0315349.s003.docx]

**Responses to Editor and Reviewers**

Manuscript ID: PONE-D-23-29808

Title: Analysis of influencing factors of review efficiency of multidisciplinary scientific research projects based on improved DEMATEL model

Dear Editor and Reviewers,

We would like to express our sincere appreciation for the time and effort the reviewers have taken in evaluating our manuscript, "*Analysis of influencing factors of review efficiency of multidisciplinary scientific research projects based on improved DEMATEL model*". We find the reviewers' comments insightful and helpful in enhancing the quality of the manuscript.

To address the reviewers’ comments, we have carefully revised our manuscript and believe that these modifications have substantially improved the manuscript. We have provided a point-by-point response to the comments below. In addition, we have uploaded a marked copy of the manuscript as a supplemental file to clearly highlight the changes made in response to the reviewers' comments.

Thank you once again for considering our work for publication in *PLoS One*. We believe that our manuscript has been significantly improved and hope that it is now suitable for publication.

I look forward to hearing from you regarding our submission.

Sincerely yours,

Yana Xiao

Senior engineer/Director

Guangdong Science & Technology Infrastructure Center

Email: ynaxiao@163.com

**Response to Editor**

**Comment 1:** In my opinion change of the rating scale is a too minor change to address the method as an ‘improved’ one. It has been done before, for example in this study: Dytczak M, Ginda G (2016) DEMATEL-based ranking approaches — Procedury rangowania w metodzie DEMATEL. Zeszyty Naukowe Wyższej Szkoły Bankowej we Wrocławiu 2016 vol. 16 no. 3 Applicability of quantitative methods to economics, finance, and management, s. 191–202. Please, refer to the method as DEMATEL with a 5-point scale as the core algorithm is not changed in any way.

**Response:** Thank you for your insightful comment. We acknowledge that the change to a 5-point scale is a recognized modification and does not fundamentally alter the core DEMATEL algorithm. We have revised our title and manuscript accordingly to accurately describe the methodology used. The revised title is "Analysis of influencing factors on review efficiency of multidisciplinary scientific research projects using DEMATEL with a 5-point scale".

**Response to Reviewer #1**

**Comment 1:** Please reorganize the manuscript at the journal request. Please change the reference format.

**Response:** Thank you for your reminder. We have revised the entire article and adjusted the manuscript to the journal's standards.

**Comment 2:** The language of this manuscript is very bad and needs help from native speakers.

**Response:** Thank you for your reminder. We invited a native English speaker to polish the entire article to improve the English expression of this manuscript. We have uploaded tracking of the changes as an additional attachment, and we hope our changes address your concerns.

**Comment 3:** The title of the manuscript should fully demonstrate the content of this study and the relevant subjects.

**Response:** Thank you for your valuable suggestion. We have revised the title to better reflect the content and scope of our study. The new title is " Analysis of influencing factors on review efficiency of multidisciplinary scientific research projects using DEMATEL with a 5-point scale." This title highlights our focus on improving review efficiency, the multidisciplinary nature of the projects studied, and the use of a DEMATEL model as our primary analytical tool.

**Comment 4:** Abstracts should include the purpose and findings of the study.

**Response:** Thanks for your suggestion. We have revised the abstract to clearly state the purpose and findings of our study. The revised abstract is:

"*In the wake of advancing technology and the convergence of diverse disciplines, collaborative research across academic sectors has become instrumental in fostering innovation and tackling multifaceted challenges. The inherent complexity of such multidisciplinary endeavors, characterized by a myriad of research trajectories and a spectrum of expertise, poses significant challenges to effective review. This study aims to identify and analyze the factors influencing the review efficiency of multidisciplinary scientific research projects to ensure their smooth development and improved quality. To address this challenge, we employ the Decision Making Trial and Evaluation Laboratory (DEMATEL) method with a 5-point scale. First, we introduce an indexing framework to systematically identify factors influencing the appraisal of multidisciplinary efforts. This framework is then complemented by expert-driven questionnaires, harnessing domain-specific insights to ascertain the significance and interconnectedness of these factors. Using the DEMATEL method, we distill the data to identify key influencing factors that enhance review efficiency for multidisciplinary projects. Our findings provide pragmatic strategies and policy guidance, equipping institutional bodies and program leads with tools to refine the review process of multidisciplinary scientific research projects.*"

**Comment 5:** Introduction. This a very vague statement. These sentences do not provide any information on how the concept could be conceptualized? This section should explain the study's context and research objective. Furthermore, the research gap needs to be narrowed after analyzing the previous studies. The research method is not adequately explained in the first section. Introduction, what authors wanted to convey. Here author must build research gap following the previous studies.

**Response:** Thanks for your constructive comment. We agree that the introduction should provide a clearer context, outline the research objective, highlight the research gap after analyzing previous studies, and briefly explain the research method. Below is the revised introduction addressing these points. All changes are marked in different colors in the document, please see the manuscript with change tracked.

**Comment 6:** The manuscript does not answer the following concerns: Why is it timeliness to explore such a study? What makes this study different from the previously published studies? Are there any similarly findings in line with the previously published studies? Are the findings different from prior academic studies that were conducted elsewhere, if any? For example, information innovation and innovation network, what it requires, what are the new technologies, some recent issue highlights the importance. See the following:

[1] Digital green value co-creation behavior, digital green network embedding and digital green innovation performance: moderating effects of digital green network fragmentation.

[2] Enhancing digital innovation for the sustainable transformation of manufacturing industry: a pressure-state-response system framework to perceptions of digital green innovation and its performance for green and intelligent manufacturing.

[3] Developing a Conceptual Partner Selection Framework: Digital Green Innovation Management of Prefabricated Construction Enterprises for Sustainable Urban Development.

**Response:** Thanks for your detailed and constructive comment. We recognize the importance of addressing these concerns to provide a comprehensive context for our study. Below is the revised introduction, incorporating answers to these questions. All changes are marked in different colors in the document, please see the manuscript with change tracked.

**Comment 7:** Methodology: Model. I suggest authors here build your main heading on Research and data methodology. Clearly explain the model building process, and what previous studies have used similar models (model testing approach).

**Response:** Thanks for your comment. We supplemented the description of the model establishment details in the revision as follows:

“***3.3 Model building process***

*In this study, we employ the DEMATEL method with a 5-point scale to analyze the factors influencing the review efficiency of multidisciplinary scientific research projects. The DEMATEL method is a widely recognized tool for visualizing and quantifying the causal relationships among complex system components. It has been previously applied in various fields such as supply chain management, risk assessment, and technology planning [37,43].*

***Step 1. Indexing framework development***

***Step 2. Expert Consultation***

***Step 3. Expert-driven questionnaires***

***Step 4. DEMATEL analysis***”

The additional details of the model construction make this manuscript more complete. Thank you again for your suggestions and we hope that our revisions have addressed your concerns.

**Comment 8:** There is no flow in the text. It partly depends on the lack of proofreading but also on the fact that many statements and claims are made without being followed up by a clear and logical discussion. It is especially problematic in the Introduction that brings up a number of findings from different areas without linking them together.

**Response:** Thanks for your comment. We have revised the Introduction to ensure better flow and logical discussion, linking the findings from different areas cohesively. The revised Introduction with improved coherence and logical connections are attached to the Manuscript with change tracked. We hope this revised introduction provides a clearer and more cohesive context, linking the findings from different areas logically and ensuring better flow in the text. Thanks again for your valuable comment.

**Comment 9:** Please make sure your conclusions' section underscores the scientific value-added of your paper, and/or the applicability of your findings/results. Highlight the novelty of your study.

**Response:** Thanks for your comment. We have revised the Conclusion section to underscore the scientific value-added of our paper, highlight the applicability of our findings/results, and emphasize the novelty of our study. The new Conclusion section contains Managerial implication, Practical/social implications, Managerial implication, and Future directions.

**Comment 10:** In addition to summarizing the actions taken and results, please strengthen the explanation of their significance. It is recommended to use quantitative reasoning comparing with appropriate benchmarks, especially those stemming from previous work. See the following: Developing a Conceptual Partner Matching Framework for Digital Green Innovation of Agricultural High-End Equipment Manufacturing System Toward Agriculture 5.0: A Novel Niche Field Model Combined With Fuzzy VIKOR

**Response:** Thanks for your comment. We have revised the Conclusion and Discussion section to strengthen the explanation of the significance of our findings. We included quantitative reasoning and comparisons with appropriate benchmarks from previous studies to illustrate the importance of our results. The revised discussion and conclusion highlight the scientific value-added, applicability of findings, novelty of the study, and provides a detailed quantitative comparison to underscore the significance of our research.

**Comment 11:** More importantly, the choice of the variables should be explained in light of the theory and the prior literature on the topic. The arguments are simply relationships and causes very close to the replication of many studies dealing with the same thing.

**Response:** Thanks for your comment. We have revised the manuscript to explain the choice of variables in light of existing theories and prior literature, ensuring that the arguments are grounded in a solid theoretical framework. All changes are marked in different colors in the document, please see the manuscript with change tracked.

**Comment 12:** The authors should emphasize the important role of digital technology in industrial structure upgrading in future research. Some recent issue highlights the importance: The Interaction Mechanism and Dynamic Evolution of Digital Green Innovation in the Integrated Green Building Supply Chain.

**Response:** Thanks for your comment. We have revised the Introduction of the manuscript to emphasize the important role of digital technology in industrial structure upgrading as follows:

“*Digital technology plays a crucial role in the industrial structure upgrading process. It facilitates the integration of advanced technologies, enhances productivity, and drives innovation across various sectors. The interaction mechanism and dynamic evolution of digital green innovation in integrated supply chains, such as in green building, highlight the transformative potential of digital technology. For example, digital green innovation can streamline operations, reduce environmental impact, and foster sustainable development by optimizing resource use and enhancing efficiency.*”

**Comment 13:** Please consider this structure for manuscript final part.

-Discussion

-Conclusion

-Managerial Implication

-Practical/Social Implications

**Response:** Thanks for your comment. In the revised manuscript we have reorganized the conclusion chapter and restructured our article according to your suggested structure and hope that our revisions can address your concerns.

**Comment 14:** Discussion needs to be a coherent and cohesive set of arguments that take us beyond this study in particular, and help us see the relevance of what authors have proposed. Authors should create an independent “Discussion” section. Author need to contextualize the findings in the literature, and need to be explicit about the added value of your study towards that literature. Also other studies should be cited to increase the theoretical background of each of the method used. Findings should be contextualized in the literature and should be explicit about the added value of the study towards the literature (New Energy-Driven Construction Industry: Digital Green Innovation Investment Project Selection of Photovoltaic Building Materials Enterprises Using an Integrated Fuzzy Decision Approach). Limitations and future research.

**Response:** Thanks for your comment. In the revised manuscript we have reorganized the “Discussion” section to contextualize our findings within the existing literature, highlight the added value of our study, cite additional relevant studies, and discuss the limitations and future research directions. We hope this revised Discussion section address your concerns.

**Comment 15:** As any emprical study that use different approaches I would like to ask to introduce in the Conclusion section at least a paragraph containing the study limitations. I noticed some things in the paper but a synthesis of statements related to how the study is useful (or partially useful, since are required certain further analysis) and helps potential interested readers does not really exist. Maybe in addition to the last section of Conclusion it is beneficial to introduce a section called: Discussion.

**Response:** Thanks for your comments. We acknowledge your concerns and provide the limitations part in the revised Discussion section.

**Response to Reviewer #2**

**Comment 1:** The reader of the paper can anticipate a more detailed account of the way the researchers conducted their analyses. Unfortunately, the revised version of the manuscript offers limited additional specifics in this regard.

**Response:** Thanks for your suggestions. We have provided a more detailed account of our methodology and analysis process in the revised manuscript to address this concern. All changes are marked in different colors in the document, please see the manuscript with change tracked.

**Comment 2:** Regarding the comment about the collection of documents used to identify indicators, the authors simply cited in the revised manuscript the names of the large publication databases referenced by the reviewer (“Elsevier’s ScienceDirect, SpringerLink, open access publications”). The number of documents reviewed, types of publication sources utilized, and the method of narrowing down the set of documents in subsequent research stages are not disclosed. The reader is left without a clear understanding of how the relevant literature was fished out from the ocean of publications available nowadays.

**Response:** Thanks for your suggestions. We have revised a more detailed explanation of the document collection process, including the number of documents reviewed, types of publication sources utilized, and the method of narrowing down the set of documents. All changes are marked in different colors in the document, please see the manuscript with change tracked.

**Comment 3:** The authors of the study revealed that the experts involved were predominantly from Pakistan, yet left the reader unaware of the reasoning behind this particular choice of the experts’ background. That appears quite perplexing, considering the paper is meant to tackle considerable challenges of reviewing complex interdisciplinary projects, which are known for their “myriad of research trajectories and a spectrum of expertise”. Once more, there is a lack of specific details regarding the number of experts and their characteristics are rather general (“from diverse backgrounds”, “well-qualified”, “served on various selection committees”).

**Response:** Thanks for your suggestions. We acknowledge the need to provide more specific details regarding the selection of experts and their backgrounds. We will revise the manuscript to include the reasoning behind the choice of experts, their number, and their detailed characteristics.

**Comment 4:** The authors addressed the comments about the type of research projects and the type of review that are of interest to their paper and explained that their main focus is on applied research rather than theoretical research as well as on the interim research review rather than the research project review at the stage of the proposal submission. However, these points were not explicitly stated in the main body of the paper.

**Response:** Thanks for your suggestions. We revised the manuscript to explicitly state that our main focus is on applied research rather than theoretical research and on the interim research review rather than the research project review at the stage of the proposal submission.

**Comment 5:** The explanation for why the factor F8 “Review methodology” is included does not fully address the doubts about the analysis's somewhat circular reasoning. In a general sense, methodology refers to the set of procedures or techniques used to identify, select, process, and analyze information pertaining to a particular subject, so the research project review methodology can be seen to encompass other factors listed in the paper. It is possible that the authors intended to refer to some particular aspect of the review methodology, but they did not make it clear.

**Response:** Thanks for your suggestions. We acknowledge the need to clarify the inclusion and specific aspect of the “Review methodology” (F8) in our analysis. We will revise the manuscript to address this concern by specifying the particular aspect of the review methodology that we focused on.

**Comment 6:** The authors do not provide an explanation for why the reviewer's expertise had the least effect on the review efficiency, a finding that goes totally against expectations.

**Response:** Thanks for your suggestions. We acknowledge the need to provide an explanation for why the reviewer's expertise had the least effect on review efficiency, which is indeed counterintuitive. We will revise the manuscript to address this finding and provide a rationale based on our data and contextual analysis. All changes are marked in different colors in the document, please see the manuscript with change tracked.
