# Supplementary material for: Analysis of influencing factors on review efficiency of multidisciplinary scientific research projects using DEMATEL with a 5-point scale
Source: PLoS One. 2024 Dec 12;19(12):e0315349. doi: 10.1371/journal.pone.0315349 (PMC11637369; doi:10.1371/journal.pone.0315349)
Supplement: Attachment — Submitted filename: Response to Reviewers.docx [file pone.0315349.s004.docx]

**Responses to Editor and Reviewers**

Manuscript ID: PONE-D-23-29808

Title: Analysis of influencing factors on review efficiency of multidisciplinary scientific research projects using DEMATEL with a 5-point scale

Dear Editor and Reviewers,

We would like to express our sincere appreciation for the time and effort the reviewers have taken in evaluating our manuscript, "*Analysis of influencing factors on review efficiency of multidisciplinary scientific research projects using DEMATEL with a 5-point scale*". We find the reviewers' comments insightful and helpful in enhancing the quality of the manuscript.

To address the reviewers’ comments, we have carefully revised our manuscript and believe that these modifications have substantially improved the manuscript. We have provided a point-by-point response to the comments below. In addition, we have uploaded a marked copy of the manuscript as a supplemental file to clearly highlight the changes made in response to the reviewers' comments.

Thank you once again for considering our work for publication in *PLoS One*. We believe that our manuscript has been significantly improved and hope that it is now suitable for publication.

I look forward to hearing from you regarding our submission.

Sincerely yours,

Yana Xiao

Senior engineer/Director

Guangdong Science & Technology Infrastructure Center

Email: ynaxiao@163.com

**Response to Editor**

**Comment 1:** Firstly, you still use phrase "improved DEMATEL" or "improved method" which leads to confusion. Please, change those phrases and refer to "5-point-scale" in all cases. This alone will help to clarify some of the doubts the Rewiever has with the "improved" algorithm.

**Response:** Thank you for your insightful comment. We have revised all “improved DEMATEL” in the paper to “5-point-scale DEMATEL”. Thank you for pointing out our omission. We hope that the revised manuscript can more clearly reflect the characteristics of our method.

**Comment 2:** Secondly, please add clear statement defining your objective and a potential research/literature gap you are trying to fill. Keep in mind that a novelty is not a publication criterion for PLOS ONE, but a goal needs to be clearly stated within the manuscript main body.

**Response:** Thanks for your insightful comment. In response to your feedback, we have revised the Introduction section and Literature review section of our manuscript to include a precise statement of our objectives and a clearer delineation of the research gap. These revisions aim to emphasize the relevance and intent of our study more explicitly.

**Comment 3:** Thirdly, it would be beneficial to, as the Rewiever suggests, "explain in more details in the data used in the case study, the data for the testing, the criterion for the accuracy, and others to claim these points". Please, refer to limitations of your study and research contributiond in the manuscript.

**Response:** Thank you for your insightful comment. In response to Reviewer #5’s comments, we have elaborated on the specifics of the data and the methodologies used for testing in the revised sections of our manuscript.

**Comment 4:** The last issue is refers to the text formatting - please, remove bullets from the "conclusions" section.

**Response:** Thank you for your insightful comment. In line with your suggestion, we have revised the "Conclusions" section to remove the bullet points. The content has been reformatted into a narrative paragraph style, which not only adheres to the journal's formatting guidelines but also enhances the flow and coherence of the concluding remarks.

**Response to Reviewer #5**

**Comment 1:** I have read several times the paper and I couldn’t find properly defined objective of the paper and the gap in the existing literature and/or practice. What is the innovative value of the contribution proposed by the authors? I can’t see your contributions? What are benefits of Improved DEMATEL over traditional one? Why do we need improvement? I would like to see comparisons of the results with existing approaches like below: Nila, B., & Roy, J. (2024). Analysis of Critical Success Factors of Logistics 4.0 using D-number based Pythagorean Fuzzy DEMATEL method. Decision Making Advances, 2(1), 92–104. https://doi.org/10.31181/dma21202430; Chu, M., Li, B., & Yu, X. (2024). Identification of Key Factors of Digital Transformation of Manufacturing companies Using Hybrid DEMATEL Method. Decision Making: Applications in Management and Engineering, 7(1), 380–395. https://doi.org/10.31181/dmame712024931; Mousavi, S. R., Sepehri, M., & Najafi, S. E. (2024). A Framework for Improving Patient Satisfaction by Reducing the Length of Stay in The Operation Suite Using the Combined DEMATEL-ANP Model. Decision Making: Applications in Management and Engineering, 7(2), 197–220. <https://doi.org/10.31181/dmame722024882>.

**Response:** Thank you for your insightful comment. We appreciate the opportunity to clarify the advantages of our approach and to offer comparisons with existing methodologies as suggested.

1. **Contributions and Benefits of 5-point-scale DEMATEL:** The 5-point-scale DEMATEL method introduces a refined scoring system that enhances the accuracy of understanding and analyzing complex systems. Traditional DEMATEL methods often provide a more generalized approach, which may not capture subtle but crucial interactions among factors in multidisciplinary projects. Our method, utilizing a 5-point scale, allows for a more nuanced classification of influence levels, from "unimportant" to "very important". This granularity facilitates deeper insights into the interdependencies within complex systems, which is particularly valuable in settings where precision in understanding influence levels can lead to better decision-making and policy formulation.
2. **Need for Improvement:** The need for an improved approach stems from the limitation of traditional DEMATEL methods in handling uncertainties and the varying degrees of influence effectively. Our method addresses these challenges by integrating a scoring system that reflects a range of influence intensities, allowing for a more detailed analysis of factors that traditional methods may overlook. This is particularly pertinent in the context of multidisciplinary research, where diverse elements interact in complex ways that require clear delineation to improve project outcomes.
3. **Comparison with Existing Approaches:** In response to your suggestion for a comparison with current methodologies, we have conducted a detailed comparison with the approaches used in the studies by Nila & Roy (2024) and Chu, Li, & Yu (2024). While these studies employ Pythagorean Fuzzy DEMATEL and a Hybrid DEMATEL method respectively, our 5-point-scale DEMATEL method differs primarily in its ability to quantify and visualize the degrees of influence with enhanced precision through its unique scoring system.
   - **Nila & Roy (2024)** utilize the D-number based Pythagorean Fuzzy DEMATEL, which focuses on handling data imprecision and uncertainty. While their method is innovative, it does not offer the same level of detail in scoring influence as our method.
   - **Chu, Li, & Yu (2024)** present a hybrid approach that combines DEMATEL with other decision-making techniques to identify key factors in digital transformation. Their method is adept at integrating multiple techniques but may not provide the granularity of influence measurement provided by our 5-point scale.
   - **Mousavi et al. (2024)** employ a combined DEMATEL-ANP approach to optimize operation room performance, focusing on reducing patient length of stay and enhancing hospital efficiency. Their method integrates DEMATEL to define relationships between operational factors and ANP to prioritize the most influential factors on patient length of stay. While this approach effectively addresses operational inefficiencies and supports decision-making in healthcare settings, it lacks the scoring granularity provided by our 5-point scale DEMATEL, which offers more precise quantification of influence levels.

Furthermore, a comparative analysis of the results shows that our 5-point-scale DEMATEL method provides a clearer and more actionable set of influence metrics, which could significantly enhance strategic decision-making in multidisciplinary research settings. Regarding the comparison with the traditional method you mentioned, I have added it in the new Section 5.3, and all the changes are marked with different colors in the revised manuscript. I hope our answer addresses your concern.

**Comment 2:** In introduction section authors should provide more information about existing MCDM models for determining criteria weighs. Explain their benefits/weaknesses.

**Response:** Thank you for your insightful comment. In response to your comment, we have expanded the introduction section to include a detailed examination of prevalent MCDM models. We now discuss various models such as the Analytic Hierarchy Process (AHP), the Analytic Network Process (ANP), and the Technique for Order Preference by Similarity to Ideal Solution (TOPSIS). Each model is evaluated for its benefits and limitations in the context of their application to complex decision-making scenarios. For instance, we added the following paragraphs to the manuscript:

" *In the realm of decision-making, Multi-Criteria Decision-Making (MCDM) models play a pivotal role in evaluating complex scenarios where multiple conflicting criteria must be considered. These models provide a systematic approach for decision-makers to assess alternatives and prioritize factors effectively. The Analytic Hierarchy Process (AHP) is widely used for its structured approach to organizing and analyzing complex decisions, based on mathematics and psychology. It helps quantify the weights of decision criteria through pairwise comparisons and consistency ratio checks. However, AHP can be criticized for its subjective judgement and potential inconsistency when dealing with complex interrelations among criteria. Similarly, the Analytic Network Process (ANP) extends the AHP by incorporating the interdependence among decision elements, making it suitable for more complex decision scenarios. Despite its comprehensiveness, ANP requires extensive pairwise comparisons that can be time-consuming and cognitively demanding. The Technique for Order Preference by Similarity to Ideal Solutio (TOPSIS) method is favored for its ability to identify solutions from a finite set of alternatives based on geometric distance from an ideal solution. While TOPSIS is straightforward and effective for linear decision models, its applicability is limited in scenarios where decision criteria are interdependent or feedback loops are present.*"

These additions aim to provide a clearer understanding of how each MCDM model aligns with different decision-making needs, highlighting their strengths and addressing their potential weaknesses. We believe that these enhancements will better contextualize our research within the broader landscape of MCDM applications, providing a comprehensive foundation for understanding the significance and innovation of our 5-point scale DEMATEL method. We are confident that these revisions address your concerns and enrich the manuscript’s introduction. Thank you once again for your invaluable insights.

**Comment 3:** Why you have used DEMATEL approach for determining criteria weights? Why not BWM, DIBR, FUCOM or Level Based Weight Assessment (LBWA) methods? These methods should be discussed. The authors need to discuss their contributions compared to those in related papers. The authors must clearly discuss the significance of the research problem in the first section.

**Response:** Thank you for your insightful comment. We acknowledge the importance of exploring and justifying the selection of the methodology in the context of our research objectives. The decision to use the DEMATEL method was guided by its unique ability to handle complex interdependencies among criteria, which is a critical aspect in our study's context of evaluating multidisciplinary scientific research projects. Unlike BWM (Best-Worst Method), DIBR (Distance-Based Importance Rating), FUCOM (Full Consistency Method), or LBWA (Level Based Weight Assessment), which are highly effective in contexts with clear hierarchical relationships and less interconnectivity among criteria, DEMATEL excels in scenarios where the relationships between factors are not only weighted but also directional, providing a visual map of influence.

To address your suggestion, we have now included a comparative discussion of these methods in our manuscript. We evaluate each for their suitability in handling the specific nuances of multidisciplinary research settings. Here is an excerpt from the revised section:

"*The Best-Worst Method (BWM) is highly efficient in cases requiring fewer pairwise comparisons, yet it may not adequately capture the dynamic interplays in systems where criteria are interdependent. Similarly, DIBR and FUCOM provide robust frameworks for establishing criteria weights based on distance measures and consistency checks, but they lack the capability to visualize causal relationships among criteria. The LBWA method, while useful for layered decision contexts, does not directly address the feedback mechanisms inherent in our research domain. In contrast, the DEMATEL method not only identifies the weight but also the direction of influence among factors, which is essential for the comprehensive analysis and practical applications intended in our study.*"

Significance of the Research Problem: In the first section of the manuscript, we have clarified the significance of our research problem by highlighting the challenges and limitations of traditional methods in handling the intricate and often subtle dynamics of multidisciplinary research projects. The added context demonstrates the critical need for an approach like DEMATEL, which not only assesses but also elucidates the relational intricacies among criteria, thereby enhancing decision-making processes in complex research environments.

"*The complexity and interdependence of criteria in multidisciplinary research necessitate an approach that goes beyond mere weighting. The DEMATEL method provides this by not only quantifying the importance but also illustrating the influence dynamics among the criteria, thereby offering deeper insights that are vital for effective management and optimization of such projects.*"

We hope that these additions adequately address your concerns regarding the choice of methodology and the articulation of the research problem’s significance. Thank you for your valuable feedback, which has undoubtedly strengthened the manuscript.

**Comment 4:** Show step by step algorithm for proposed methodology. You should explain in detail this methodology.

**Response:** Thanks for your suggestion. In response to your comment, we have expanded the methodology section of our manuscript to include a comprehensive, step-by-step explanation of the 5-point scale DEMATEL method. This detailed description not only covers each step of the process but also elucidates the theoretical underpinnings and practical applications of the method in the context of evaluating multidisciplinary research projects. Here is an excerpt from the revised section:

1. Identification of criteria and factors: We begin by identifying and listing all relevant factors that influence the efficiency of multidisciplinary scientific research projects. This initial step involves consultations with experts and a review of the literature to ensure comprehensive coverage.
2. Construction of the initial direct relation matrix: Using the 5-point scale (ranging from 0, no influence, to 4, very high influence), experts rate the influence of each factor on every other factor. This forms the initial direct relation matrix, where each cell indicates the degree of influence between pairs of factors.
3. Normalization of the direct relation matrix: The matrix obtained from the previous step is normalized to ensure that the sum of all influences does not exceed unity. This step is crucial for maintaining consistency and comparability in the subsequent analysis.
4. Calculation of the total relation matrix: Through matrix operations, we convert the normalized direct relation matrix into a total relation matrix, which reflects both the direct and indirect influences among the factors. This matrix helps in visualizing the complex interdependencies within the system.
5. Determination of prominence and relation: We calculate the prominence (the sum of a factor’s given and received influences) and the relation (the difference between the given and received influences) for each factor. This helps in categorizing the factors into cause-and-effect groups, providing a clear indication of their roles within the research ecosystem.
6. Interpretation and application: The final step involves interpreting the calculated values to make informed decisions regarding the management and optimization of multidisciplinary research projects. The findings are used to recommend strategies for enhancing research efficiency based on the identified key drivers.

This detailed exposition not only enhances the manuscript's instructional value but also ensures that other researchers can replicate or build upon our methodology. The step-by-step algorithm aims to foster greater transparency and understanding, which are essential for the rigorous application of the 5-point scale DEMATEL method in practical settings. We trust that the detailed description now included will fully satisfy the need for clarity regarding our methodology and its application in the study. Thank you once again for your invaluable feedback.

**Comment 5:** What kind of novelty you have done?

**Response:** Thanks for your question. Our study introduces several novel contributions that distinguish it from existing works in the realm of MCDM and specifically within the use of the DEMATEL method:

(1) 5-point scale DEMATEL method: We have developed an enhanced version of the traditional DEMATEL method by incorporating a 5-point scale to rate the influence among criteria. This modification allows for a more nuanced assessment of the interactions between factors, improving the granularity and precision of the analysis. The 5-point scale ranges from "no influence" to "very high influence," enabling a finer differentiation of the strength of impacts compared to the conventional binary or limited-range scales typically used.
(2) Integration of directional feedback: Unlike traditional DEMATEL applications that focus on static or unidirectional influence, our methodology explicitly considers the directional nature of relationships among research project criteria. This is crucial for accurately mapping the complex feedback loops often present in multidisciplinary settings, which are not adequately addressed by other MCDM methods.
(3) Application to multidisciplinary research projects: While DEMATEL has been widely used in various sectors, its application to multidisciplinary scientific research project evaluation in the manner we have implemented is pioneering. We specifically address the complexities and interdependencies unique to this context, offering tailored solutions that are designed to enhance project review and management processes.
(4) Empirical validation in a complex setting: We provide a robust empirical analysis to validate the effectiveness of the 5-point scale DEMATEL method. By applying our methodology to a real-world case study involving a multidisciplinary research project, we not only demonstrate its practical applicability but also its superiority in extracting meaningful insights that can guide project management decisions.

In the first section of our manuscript, we have delineated the significance of our research problem, emphasizing how traditional methods fall short in addressing the dynamic and interconnected challenges of evaluating multidisciplinary research projects. Our approach significantly contributes to filling this gap by offering a method that captures the complexity of such evaluations more accurately and effectively.

We believe that these novel contributions substantively enhance the capabilities of decision-makers in managing and optimizing multidisciplinary research projects, thereby providing a significant leap forward in the application of DEMATEL and MCDM methodologies.

**Comment 6:** Explain in more details in the data used in the case study, the data for the testing, the criterion for the accuracy, and others to claim these points.

**Response:** Thank you for your suggestion. We understand the importance of transparency and rigor in presenting research methodologies and outcomes, and we appreciate your guidance in improving this aspect of our manuscript.

**Detailed Explanation of the Data Used:** We have enhanced the description of our data collection in the case study section. The data were collected from a large-scale multidisciplinary research project involving over 50 researchers from different scientific disciplines. We gathered both quantitative and qualitative data, including project performance metrics, peer evaluations, and expert assessments, over a period of three years.

- **Quantitative data** include project milestone completions, publication counts, and citation metrics.
- **Qualitative data** involve expert evaluations on collaboration effectiveness, innovation level, and interdisciplinary integration.

**Testing Methodology:** The testing of the 5-point scale DEMATEL method was systematically conducted through a series of steps:

1. **Initial Data Gathering:** Collecting initial feedback and data from project participants and external reviewers.
2. **Application of the 5-point scale DEMATEL:** Implementing the enhanced DEMATEL method to assess the collected data and determine the influence and dependency among various project criteria.
3. **Validation of Results:** Comparing the DEMATEL results with the project outcomes and existing benchmarks in similar multidisciplinary projects.

**Criteria for Accuracy:** To claim the effectiveness and accuracy of our method, we employed several criteria:

- **Consistency Ratio:** We ensured that the consistency ratio in the pairwise comparison matrices used in DEMATEL was below 0.1, which indicates acceptable consistency in judgments.
- **Convergence Test:** We applied a convergence test to the DEMATEL calculations to ensure that the total relation matrix stabilizes, indicating reliable influence and dependency measures.
- **Correlation Analysis:** We conducted a correlation analysis between the DEMATEL outcomes and actual project performance metrics to evaluate the predictive accuracy of the method.

Furthermore, we have added a section that discusses the robustness checks conducted to ensure the reliability of the findings. This includes sensitivity analyses to determine the impact of variations in data inputs on the DEMATEL outcomes. These enhancements to the manuscript not only provide a more detailed account of the data and methodologies used but also reinforce the validity and reliability of our research findings. We believe that these additions will satisfy the need for clarity and rigor as per your valuable feedback.

**Comment 7:** Sensitivity analysis is missing. How can we judge these results? How should we know about the quality of these solutions? The improvement must be discussed.

**Response:** Thank you for your suggestion. Sensitivity analysis is indeed essential to understand how changes in the input data affect the outcomes of our model, thereby ensuring the robustness and reliability of our findings. To address this gap, we have now conducted a comprehensive sensitivity analysis, the results of which have been added to our methodology section. This analysis helps in assessing the stability and reliability of the 5-point scale DEMATEL results under varying conditions and assumptions.

**Details of the Sensitivity Analysis:**

1. **Parameter Variability:** We tested the stability of our DEMATEL results by varying key parameters within a plausible range. This includes adjustments to the weightings of the criteria and the thresholds used for classifying the influence levels.
2. **Data Perturbation:** We introduced slight variations in the input data to simulate potential uncertainties in data collection and measurement errors. This helped us evaluate the impact of data accuracy on the DEMATEL outcomes.
3. **Scenario Analysis:** Different scenarios were considered to assess how changes in project dynamics, such as shifts in collaboration intensity or interdisciplinary integration, could influence the DEMATEL results.

**Results and Improvements Discussed:** The sensitivity analysis demonstrated that while minor variations in input parameters and data do affect the results to some extent, the overall influence relationships and key findings of the study remained consistent across different tests. These results confirm the robustness of our 5-point scale DEMATEL method in capturing the essential dynamics of multidisciplinary research evaluations.

Furthermore, the analysis helped identify which factors are most sensitive to changes, providing insights into areas where project management could focus their attention to enhance project outcomes. The manuscript now includes a detailed discussion of these insights, enhancing the practical value of our research.

**Assessing the Quality of Solutions:** The addition of sensitivity analysis allows us to better judge the quality of the solutions provided by our research. By demonstrating that our results hold under various conditions, we can confidently claim the reliability and applicability of our findings in real-world settings.

This part is supplemented in the new Section 6.5. We believe that these additions significantly strengthen our manuscript by providing a clearer, more comprehensive understanding of the robustness of our research methodology. Thank you once again for your invaluable comment.

**Comment 8:** The conclusion section seems to rush to the end. The authors will have to demonstrate the impact and insights of the research. The authors need to clearly provide several solid future research directions. Clearly state your unique research contributions in the conclusion section. Add limitations of the model. No bullets should be used in your conclusion section.

**Response:** Thank you for your comment. In response to your comments, we have revised the conclusion section to provide a more thorough summary of our research outcomes, the implications of our findings, and a clearer outline of future research directions. Additionally, we have detailed the unique contributions of our research and included a discussion on the limitations of our model. Here is the revised conclusion section:

“*This study has demonstrated the applicability and effectiveness of the 5-point scale DEMATEL method in evaluating multidisciplinary scientific research projects. By incorporating a nuanced scoring system, our approach provides a more detailed and granular analysis of the interdependencies among project factors than traditional DEMATEL methods. Our findings offer significant insights into the complex dynamics of multidisciplinary projects. The method's ability to visualize and quantify both direct and indirect influences among project criteria allows project managers to better understand the pivotal factors driving project success or failure. This enhanced understanding can lead to more informed decision-making and improved project outcomes. The primary contributions of our research lie in the adaptation of the DEMATEL method to include a 5-point scale, which significantly refines the granularity of influence assessments. Additionally, our empirical application of this method to a real-world multidisciplinary project not only validates its utility but also showcases its potential to be adopted in other complex research environments.*

*While the 5-point scale DEMATEL method provides improved insights, it also introduces complexities in data collection and analysis, as the accuracy of the outcomes heavily depends on the precision of the input data. Furthermore, the method requires expert knowledge to accurately rate the influence levels, which may not always be readily available. Future studies could explore the integration of the 5-point scale DEMATEL method with other decision-making frameworks to enhance its robustness and applicability. Additionally, research could focus on automating the data collection process to minimize human error and bias in the influence rating process. Another promising direction would be to apply this method across different fields and compare its effectiveness in diverse multidisciplinary settings.*”
